# The Neuroprotective Effect of NEUROMIDE, a Compound Bioidentical to Commensal Bacteria Metabolites

**DOI:** 10.3390/life12101529

**Published:** 2022-09-30

**Authors:** Yoonhee Seo, Hyunji Tak, Dohee Park, Hyejin Song, Sooyoung Choe, Chaehyeong Park, Byeongdeog Park

**Affiliations:** 1 Efficacy Evaluation Center, Dt & CRO, Yongin 17042, Korea; 2 Pomona College, 333 N College Way, Claremont, CA 91711, USA or; 3 Dr. Raymond Lab Inc., #301 GwanPyung 2 RO, 7-7, Daejeon 34019, Korea

**Keywords:** endocannabinoid, GPR119, CB1, neuroprotective, gut–brain axis

## Abstract

**Simple Summary:**

N-Palmitoyl serinol is a commensal bacterial metabolite that acts as an endocannabinoid CB1 and GPR119 agonist. We investigated the potential of N-Palmitoyl serinol (NEUROIMIDE) to exert its neuroprotective effects via the gut–brain axis. NEUROMIDE improves cell survival and can reduce and even prevent scopolamine-induced memory impairments and passive avoidance disorder while increasing acetylcholine levels. These results suggest that the endocannabinoid system can act via the gut–brain axis.

**Abstract:**

GPR119 is a novel cannabinoid receptor that is primarily expressed in the pancreas and gastrointestinal tract and has beneficial effects on glucose homeostasis exerted through the stimulation of GLP-1 secretion, as demonstrated in the rodent brain. GLP-1 also has important anti-inflammatory effects in chronic inflammatory diseases, including type 1 and 2 diabetes, asthma, psoriasis, and neurodegenerative disorders. Recently, there has been increasing interest in the effect of the gut microbiota on both the gut and the brain. However, few studies have examined how gut microbes affect brain health through the endocannabinoid system. NEUROMIDE is a compound that shares a bioidentical structure with certain commensal bacterial metabolites, acting as a CB1 and GPR119 agonist. In an in vitro system exposed to reactive oxygen species (ROS), pretreatment with NEUROMIDE resulted in a significant increase in cell viability. The ROS-exposed system also showed decreased acetylcholine and an increase in inflammatory cytokines such as IL-1β, changes that were counteracted in a dose-dependent manner in the NEUROMIDE treatment groups. To measure the effectiveness of NEUROMIDE in an in vivo system, we used scopolamine-treated mice as a neurodegenerative disease model and performed a series of passive avoidance tests to observe and quantify the cognitive impairment of the mice. Mice in the NEUROMIDE treatment group had increased latency time, thus indicating an improvement in their cognitive function. Furthermore, the NEUROMIDE treatment groups showed dose-dependent increases in acetylcholine along with decreases in TNF-α and IL-1β. These experiments demonstrate that NEUROMIDE can potentially be used for neuroprotection and the improvement of cognitive ability.

## 1. Introduction

N-Palmitoyl serinol is an endogenous compound belonging to the N-acylamide family. Within this family, N-arachidonoylethanolamide, anandamide (AEA) and oleoylethanolamide (OEA) are two of the most well-known endocannabinoids. AEA is an endogenous endocannabinoid reported to act as an agonist toward CB1, CB2 [1], and GPR55 [2], and OEA is an endogenous GPR119 agonist [3]. Additionally, 2-arachidonylglycerol (2-AG) has been reported as a full agonist of the CB1 and CB2 receptors [4] and a GRP119 agonist [5].

**Figure 1:**
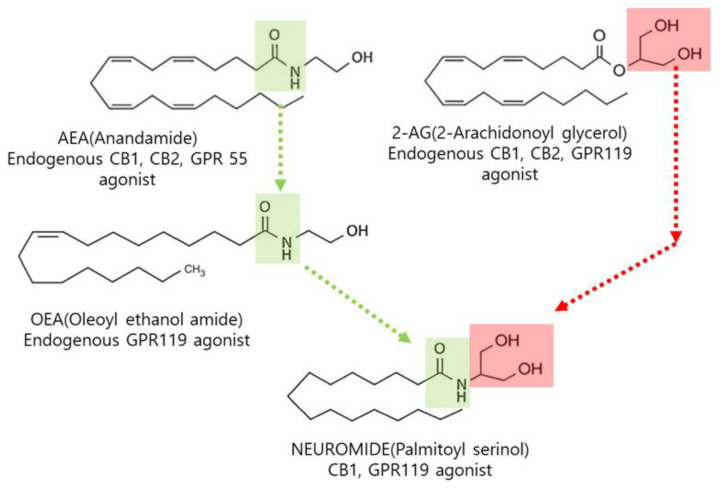
Structures of common endocannabinoids and N-palmitoyl serinol (NEUROMIDE).

Structurally, 2-AG has two hydroxyl groups derived from glycerol (Figure 1). N-Palmitoyl serinol harbors an amide bond from AEA and OEA and two hydroxyl groups from 2-AG.

N-Palmitoyl serinol is a human metabolite that is found as a component of membranes or in the cytoplasm [6,7]. In a paper published in 2000, the authors observed that the incubation of neuroblastoma cells with N-palmitoylated serinol (C16 serinol) caused apoptosis, with an IC50 of approximately 80 µM. C16 Serinol increased ceramide synthesis by 50–80% via PKCζ activation, thereby inducing apoptosis in neuroblastoma cells [8].

A report in 2017 described the extraction and characterization of N-acylamides from 128 human stool samples from 21 participants. Through comparisons with an authentic standard, N-palmitoyl serinol was confirmed as one of the N-acylamides present in the human fecal extracts [9].

It was also confirmed that *E. coli* transfected with the human microbiome N-acyl synthase hm-NAS) gene, which was derived from *Desulfitobacterium hafniense* DP7 (gastrointestinal tract, Firmicutes) and *Bacillus* sp. 2_A_57_CT2 (oral, Firmicutes), produces N-palmitoyl serinol [9]. N-Palmitoyl serinol acts as an agonist toward GPR119, an endocannabinoid receptor (EC50 = 9 µM), and has been shown to increase GLP-1 and lower blood glucose levels [9].

GPR119 is an effective target in the context of metabolic fatty liver disease [10], anti-inflammation [11], analgesia [12], colonic motility [13], gut microbiome interactions [14], homeostasis in skin sebocytes [15], and the biology of skin melanocytes [16].

In our previous study, we synthesized N-palmitoyl serinol (NEUROMIDE) and demonstrated its agonistic effects toward CB1R [17]. Activation of CB1R increased skin barrier recovery and showed effectiveness in treating inflammatory diseases such as atopic dermatitis [18]. In response to N-palmitoyl serinol, the inhibition of cannabinoid receptor CB1 as induced by the CB1 antagonist (AM-251) failed to increase levels of total ceramides and very-long-chain ceramides. These studies demonstrate that N-palmitoyl serinol stimulates the synthesis of very-long-chain ceramides (C22 and C24) and increases the total amount of ceramides via an endocannabinoid receptor CB1-dependent mechanism [19].

Transcript levels of CB1 and GPR119 are decreased in Crohn’s disease patients [20], indicating the significant role that CB1 and GPR119 agonists play in intestinal inflammation.

Previous research has shown that gastrointestinal inflammation can bidirectionally affect the brain via the gut–brain axis [21].

In this study, we examined whether N-palmitoyl serinol (NEUROMIDE) shows a neuroprotective effect under oxidative stress, by observing the levels of inflammatory cytokines in PC-12 cells and the effects on cognitive function using an in vivo mouse model.

## 2. Materials and Methods

### 2.1. Cell Culture

The PC-12 cell line was purchased from ATCC. Cells were maintained in RPMI1640 medium (Gibco BRL, Grand Island, NY, USA) supplemented with 10% horse serum, 5% fetal bovine serum (Gibco BRL), and antibiotic–antimycotic (Gibco BRL, penicillin 100 units/mL, streptomycin 100 units/mL, Amphotericin B 0.25 μg/mL in final concentration) at 37 °C in a 5% CO_2_ humidified incubator.

### 2.2. Cell Viability Test

PC-12 cells were seeded at a density of 1 × 10^5^ cells/well in a 96-well plate and cultured for 24 h. N-Palmitoyl serinol (NEUROMIDE) (Dr. Raymond Laboratories, Inc., Englewood Cliffs, NJ, USA) was applied at different concentrations with incubation for 24 h, followed by evaluation using the MTT Cell Proliferation Assay Kit (Abcam, Cambridge, UK), according to manufacturer’s instructions. The absorbance was measured at 590 nm using a microplate reader, to determine the cell viability.

### 2.3. Induction of Cell Damage

PC-12 cells were seeded at a density of 1 × 10^5^ cells/well in a 96-well plate or 1 × 10^6^ cells/well in a 6-well plate and cultured for 24 h. The medium was replaced with a serum-free medium containing NEUROMIDE and cultured for 2 h. Then, 400 µM H_2_O_2_ was added, and the cells were incubated for an additional 2 h. The well plate was centrifuged to separate the medium, and RNA was extracted from the cells. After induction of cell damage, cell viability testing, acetylcholine measurement, and qRT-PCR for IL-1β RNA in PC-12 cells were performed.

### 2.4. Cell Viability Test

Cell viability testing was performed according to the manual of the MTT Cell Proliferation Assay Kit (Abcam, Cambridge, UK). The absorbance was measured at 590 nm using a microplate reader, to determine cell viability.

### 2.5. Measurement of Acetylcholine (Ach) in PC-12 Cells

PC-12 cells were seeded and incubated at 1 × 10^6^ cells/well in a 6-well plate. After treatment with NEUROMIDE and induction of damage, the cells were centrifuged, and the amount of Ach in the supernatant was measured. Acetylcholine was measured using the Choline/Acetylcholine Quantification Kit (Biovision, Milpitas, CA, USA).

### 2.6. RNA Preparation and qRT-PCR

PC-12 cells were seeded at 1 × 10^6^ cells/well in a 6-well plate and cultured. After treatment with NEUROMIDE and the induction of damage, cells were harvested, and total RNA was extracted according to the manual of the RNA Extraction Kit (Qiagen, Hilden, Germany). Primers with the following sequences were used: IL-1β, 5′-TGACCCATGTGAGCTGAAAG-3′ (forward) and 5′-GGGATTTTGTCGTTGCTTGT-3′ (reverse); GAPDH, 5′-ACTCCCATTCTTCCACCTTTG-3′ (forward) and 5′-CCCTGTTGCTGTAGCCATATT-3′ (reverse). One-step real-time PCR was performed with 100 ng RNA using the TOPreal™ One-step RT-qPCR Kit (SYBR Green with low ROX) (Enzynomics, Daejeon, Korea), according to the manufacturer’s instructions, and analyzed using the CFX96 Touch Real-Time PCR Detection System (Bio-Rad, Hercules, CA, USA). For reverse transcription, qRT-PCR was performed at 50 °C for 30 min, followed by pre-denaturation at 95 °C for 15 min, 45 cycles of denaturation at 95 °C for 5 s, and annealing and elongation at 60 °C for 30 s. All measurements were normalized according to GAPDH using the 2^−ΔΔCT^ method based on the hydrogen-peroxide-only treatment group.

### 2.7. Animal

In passive avoidance tests, significant differences in experimental results have been observed according to sex and time of day [22]. In this experiment, only male mice were purchased and used, to avoid differences according to sex.

Male ICR mice (CrljOri:CD1, 7 weeks old) were purchased from Orient Bio Inc. (Seongnam, South Korea). Mice were kept at 19–25 °C with a 12:12 h light/dark cycle and fed with a rodent diet and water ad libitum. All animal experimental procedures were approved by the KBIOHealth Institutional Animal Care and Use Committee (approval no. KBIO-IACUC-2020-050, KBIO-IACUC-2020-111).

NEUROMIDE was dissolved in 0.5% carboxymethyl cellulose (CMC) with 4 mg/mL glyceryl stearate. The mice were divided into 5 groups of 7 animals each: a normal control group, a scopolamine group, and three NEUROMIDE (16, 40, or 100 mg/kg) pretreated groups. All treatments were orally administrated. For the untreated vehicle control group, 0.5% CMC solution with 4 mg/mL glyceryl stearate was administered. In the NEUROMIDE-pretreated groups, mice received 16, 40, or 100 mg/kg NEUROMIDE for 29 days (passive avoidance task) or 42 days (Morris water maze).

### 2.8. Morris Water Maze

The Morris water maze task was performed in a circular pool (100 cm in diameter and 50 cm in height), according to a previously published method [23]. The circular pool was divided into right-angled quarters to set an arbitrary direction. A circular platform (10 cm in diameter and 35 cm high) was placed in the center of the SW quadrant, and black paint was applied to prevent the mouse from seeing the platform (Figure 4). A visual cue was placed on the outside of the pool, facing the platform, and the platform was placed 1 cm below the water level. Data were recorded using a video camera connected to software (Smart 3.0, Barcelona Panlab, Barcelona, Spain), to perform behavioral observations. Mice underwent training to memorize the location of the platform 4 times a day (1 min/time) for 5 consecutive days. Mice were placed on the platform for 10 s then removed from the pool. Escape latency was recorded during each acquisition attempt. On day 6, all mice were subjected to a probe test without a platform and recorded for 60 s. Time spent in the target quadrant (SW) and swimming distance were measured to assess spatial learning and memory. Scopolamine was injected intraperitoneally at 1 mg/kg, 30 min prior to the acquisition test, for 5 days. Vehicle and NEUROMIDE were administered 60 min prior to the Morris water maze task. Scopolamine was not injected into the mice on day 6 (the day of the probe test).

### 2.9. Passive Avoidance Test

On the 25th day of N-palmitoyl serinol administration, the white light of the passive avoidance test box was turned off. The animal was placed in the room and allowed to acclimatize for 60 s; then, the LED light in the white room was turned on and the guillotine door was simultaneously opened. The animal was left to explore and adapt to the dark room for 180 s.

The above procedure was repeated on the 26th day, adapted so that the time from entering the dark room to turning on the light was approximately 30 s. The next day, NEUROMIDE was ingested, and 30 min later scopolamine was administered intraperitoneally at 1 mg/kg. A further 30 min after the scopolamine injection, the animal was placed in the white room, the LED light was turned on, and the guillotine door was simultaneously opened. When the animal entered the dark room, the guillotine door was closed, and 0.5 mA was applied for 3 s for electrical stimulation.

Latency time was measured 24 h after electrical stimulation. NEUROMIDE was administered 1 h before latency time measurement. The animal was placed in a white room with the light off, the LED was turned on after 10 s, and the guillotine door was simultaneously opened. The latency time was measured up to the point at which all four paws of the animal had entered the dark room. The test was terminated if the animal did not enter the dark room within 300 s.

The day after the end of the passive avoidance test, NEUROMIDE was administered, and after 30 min, scopolamine was intraperitoneally injected at a dose of 1 mg/kg. After 30 min, the hippocampus and forebrain were collected.

### 2.10. Measurement of Acetylcholine and Acetylcholine Esterase Activity in Brain Tissue

Forebrain tissues were homogenized in Choline Assay Buffer (Biovision, Milpitas, CA, USA). After centrifuging, the supernatant was used for the measurement of acetylcholine and acetylcholine esterase activity. It was measured using the Choline/Acetylcholine Quantification Kit and Acetylcholinesterase Activity Colorimetric Assay Kit (Biovision, Milpitas, CA, USA).

### 2.11. Measurement of Cytokines in Brain Tissue

Hippocampal tissues were homogenized in Tissue Protein Extraction Reagent (Biovision, Milpitas, CA, USA). After centrifuging, the supernatant was used for the measurement of TNF-α and IL-1β, employing an ELISA kit (R&D System, Minneapolis, MN, USA).

### 2.12. Statistical Analysis

Statistical analyses of all results were performed using Student’s *t*-test in SPSS (SPSS Version 21.0, IBM, Armonk, NY, USA) as previously described [19]. Data are presented as means ± SE. Independent *t*-tests for pairwise comparisons were used for data analysis. *p*-values < 0.05 were considered statistically significant. If statistical analyses had been performed using ANOVA testing, slightly different results may have been possible.

## 3. Results

### 3.1. NEUROMIDE Protects Neuronal Cells from Oxidative Stress

**Figure 2:**
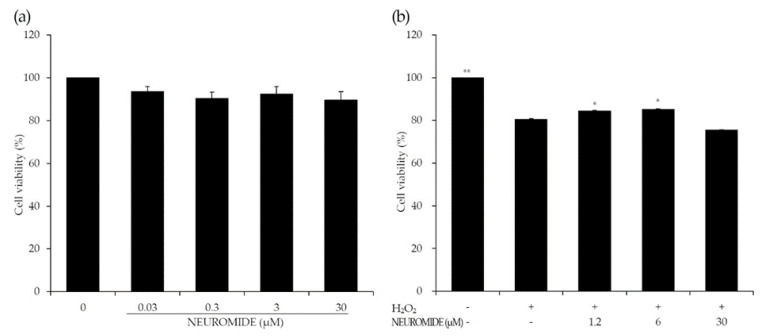
Effects of NEUROMIDE on cell viability in PC-12 cells. (**a**) PC-12 cells were treated with NEUROMIDE (0.03, 0.3, 3, and 30 μM) for 12 h. and (**b**) with serum-free medium containing NEUROMIDE (1.2, 6, and 30 μM) for 2 h, and hydrogen peroxide was added at 400 μM followed by incubation for a further 2 h. The values are the mean + SE, *n* = 3. Significant difference based on comparison with the group treated with only hydrogen peroxide, as determined by independent *t*-test: * *p* < 0.05, ** *p* < 0.01.

No statistically significant decreases in cell viability were observed for PC-12 cells treated with NEUROMIDE up to a concentration of 30 μM (Figure 2a). 

Nerve cells can be damaged due to several factors, such as loss of mitochondrial function, apoptosis induced by caspase activation, inflammation, oxidative stress, and increased acetylcholinesterase activity. We first evaluated the effects of NEUROMIDE on the PC-12 Adh cell line under oxidative stress. Cell viability was then evaluated in a hydrogen-peroxide-treated group that had been pretreated with NEUROMIDE, in comparison to the vehicle group (Figure 2b). The cell viability of the hydrogen-peroxide-treated group was reduced by 20% compared with that of the vehicle group.

Following hydrogen peroxide treatment, the 1.2 and 6 µM NEUROMIDE treatment groups showed increased cell viability compared to the group treated with only hydrogen peroxide. Cell viability in the corresponding group treated with 30 µM NEUROMIDE was slightly decreased. This assay confirmed that the groups treated with 1.2 and 6.0 µM NEUROMIDE showed statistically significant neuroprotective effects in response to subsequent hydrogen peroxide exposure.

### 3.2. Effects of NEUROMIDE on Acetylcholine and Cytokines in PC-12 Cells

Acetylcholine is required for motor function at neuromuscular junctions, but less so for learning and cognition. However, acetylcholinesterase inhibitors have long been prescribed for degenerative brain diseases such as Alzheimer’s disease. For this reason, acetylcholine was explored as a relevant biomarker in this study.

Previous research has shown that oxidative stress increases the activity of acetylcholinesterase, resulting in reduced levels of acetylcholine [24]. Therefore, preventing reductions in acetylcholine is a crucial strategy target in the field of neuroprotection.

**Figure 3:**
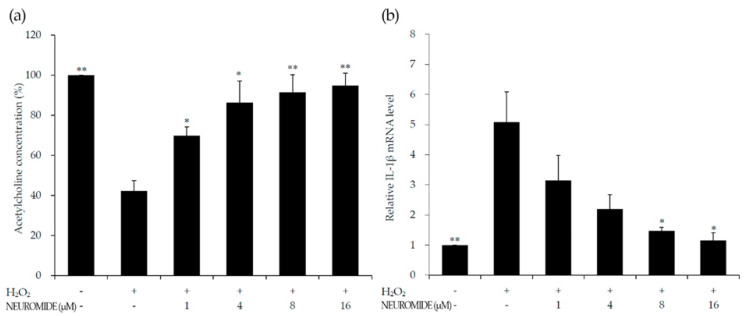
Effect of NEUROMIDE on acetylcholine and IL-1β in hydrogen-peroxide-treated PC-12 cells: (**a**) acetylcholine levels; (**b**) IL-1β mRNA expression levels. The values are means + SE, *n* = 3. Significant difference based on comparison with the group treated with only hydrogen peroxide, as determined by independent *t*-test: * *p* < 0.05, ** *p* < 0.01.

Under oxidative stress, acetylcholine concentrations were reduced to 40% of the levels in the vehicle group. The acetylcholine level increased in a NEUROMIDE-concentration-dependent manner relative to that of the group treated with only hydrogen peroxide (Figure 3a).

Oxidative stress increases the inflammatory response, e.g., via IL-1β, which is a cytokine closely linked with immunity and inflammation [25]. To test this, RT-qPCR was carried out to evaluate the effect of NEUROMIDE on the relative mRNA levels of inflammatory cytokines in response to oxidative stress.

The relative IL-1β mRNA levels in the NEUROMIDE-negative control group, i.e., treated with only hydrogen peroxide, were five times higher than those of the untreated vehicle control group (Figure 3b) and decreased in a NEUROMIDE-concentration-dependent manner. In particular, the IL-1β expression level in the 16 μM NEUROMIDE treatment group was similar to that in the vehicle group. This test confirmed that higher doses of NEUROMIDE significantly reduced IL-1β expression.

### 3.3. Effect of NEUROMIDE on Improving Spatial Memory

**Figure 4:**
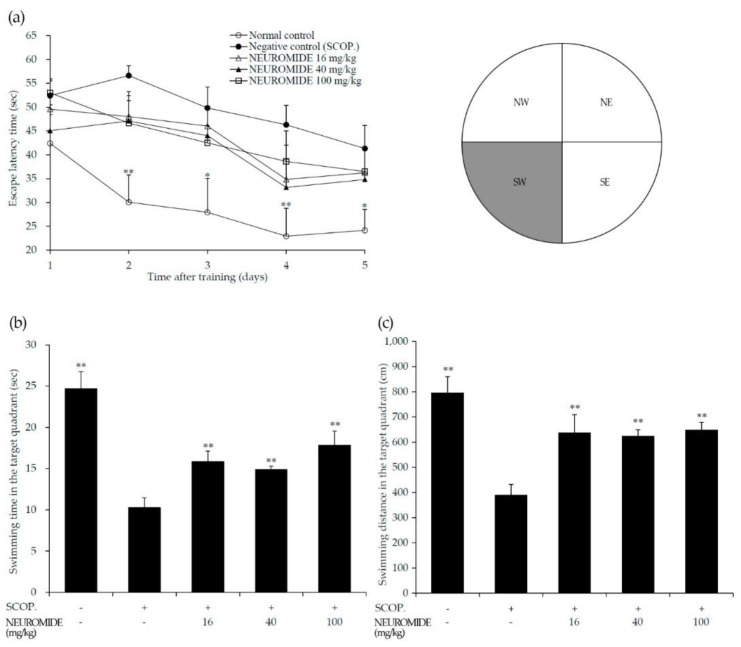
Neuroprotective effect of NEUROMIDE on SCOP-induced memory impairment in the Morris water maze task, as determined according to (**a**) escape latency time after five-day acquisition trial; (**b**) swimming time spent in the target SW quadrant on day 6; (**c**) swimming distance in the SW quadrant on day 6. SCOP: scopolamine. The values are means + SE, *n* = 7. Significant difference based on comparison with the group treated with scopolamine only, as determined by independent *t*-test: * *p* < 0.05, ** *p* < 0.01.

The Morris water maze task was performed to determine the effect of NEUROMIDE (42 days’ administration) on spatial memory following scopolamine-induced memory impairment. Training to find the hidden platform was conducted for five consecutive days. Pretreatment with NEUROMIDE reduced the time required to find the platform, compared with scopolamine-only treatment, albeit not statistically significantly (Figure 4a). Additionally, NEUROMIDE treatment significantly increased the swimming time and distance in the target quadrant in which the hidden platform was located (Figure 4b,c). This result shows that NEUROMIDE can partially alleviate scopolamine-induced spatial memory impairments.

### 3.4. Effect of NEUROMIDE on Improvements in Learning and Memory

**Figure 5:**
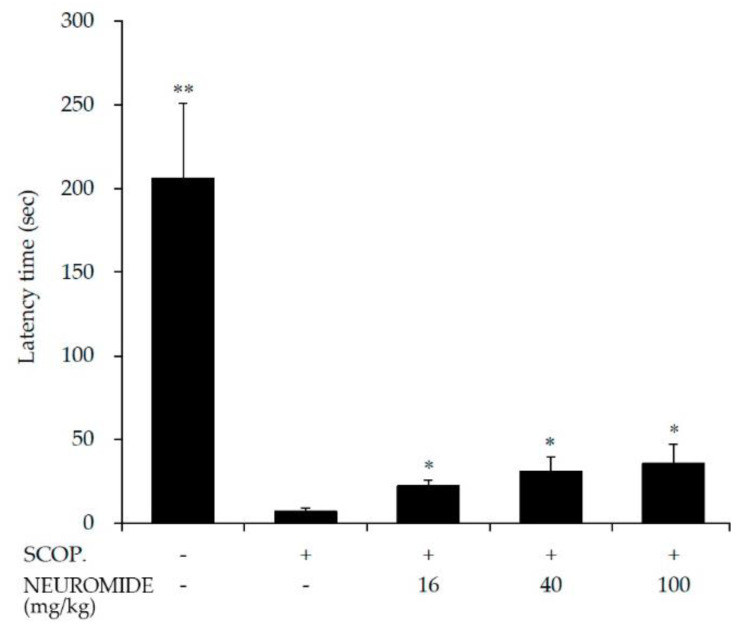
Effect of NEUROMIDE on scopolamine-induced memory impairment in a passive avoidance task. Latency time was measured when moving from the white room to the dark room on the day after administration of an electric shock in the dark room. SCOP: scopolamine. The values are means + SE, *n* = 7. Significant difference based on comparison with the group treated with scopolamine only, as determined by independent *t*-test: * *p* < 0.05, ** *p* < 0.01.

Passive avoidance tests were performed to evaluate the effect of NEUROMIDE (29 days’ administration) on the learning and memory of mice treated with scopolamine. Scopolamine-treated mice received an electric shock in a dark room. After 24 h, we measured the latency time when moving to the dark room in which the electric shock was administered. Following electric shock, scopolamine-treated mice entered the dark room (7 s) very quickly compared to untreated mice (205.9 s). The latency time was significantly increased in the corresponding NEUROMIDE-pretreated mouse groups (Figure 5). This result shows that NEUROMIDE can partially alleviate scopolamine-induced learning and memory deficits.

### 3.5. Effect of NEUROMIDE on Acetylcholine and Acetylcholine Esterase in the Forebrain

**Figure 6:**
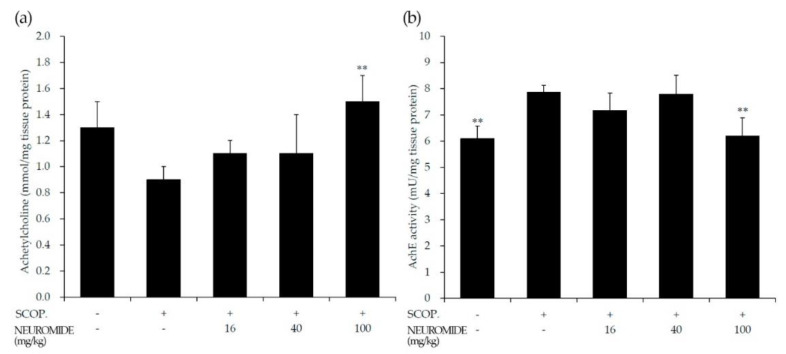
Effect of NEUROMIDE on acetylcholine and acetylcholine esterase activity in brain tissue of scopolamine-treated mice, as determined by measurements of (**a**) acetylcholine content and (**b**) acetylcholine esterase activity in the forebrain. SCOP: scopolamine. The values are means + SE, *n* = 7. Significant difference based on comparison with the group treated with scopolamine only, as determined by independent *t*-test: ** *p* < 0.01.

To evaluate the effect of NEUROMIDE at the molecular level, acetylcholine levels in the forebrain were measured after completion of the passive avoidance test. Acetylcholine was reduced in the brains of scopolamine-treated mice, compared with mice not treated with scopolamine. Acetylcholine was increased in the brains of mice pretreated with NEUROMIDE (Figure 6a). The activity of acetylcholinesterase (AchE) was increased in scopolamine-treated mice, and only to a lesser degree in mice that had received NEUROMIDE (Figure 6b). This result shows that NEUROMIDE can increase acetylcholine in the brain by inhibiting AchE activity, preventing the disruption of acetylcholine.

### 3.6. Effect of NEUROMIDE on Inflammatory Cytokines in the Hippocampus

**Figure 7:**
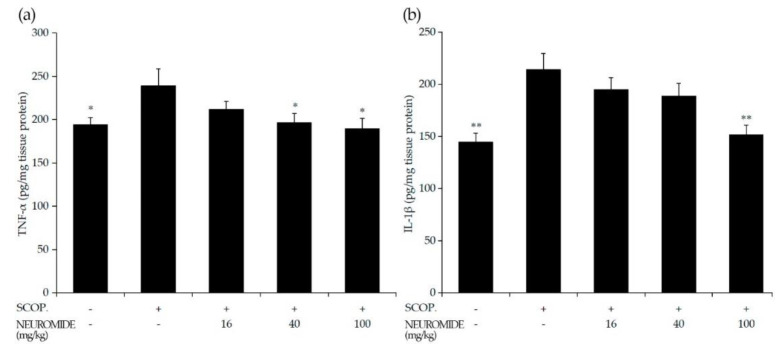
Effect of NEUROMIDE on cytokines in brain tissue of scopolamine-treated mice: (**a**) TNF-α content in hippocampus; (**b**) IL-1β content in hippocampus. SCOP: scopolamine. The values are means + SE, *n* = 7. Significant difference based on comparison with the group treated with scopolamine only, as determined by independent *t*-test: * *p* < 0.05, ** *p* < 0.01.

We quantified the cytokine content of TNF-α and IL-1β in the hippocampus (Figure 7a,b). Contents of TNF-α and IL-1β increased in scopolamine-treated mice compared with untreated mice, and decreased in mice given NEUROMIDE compared with mice treated with only scopolamine.

## 4. Discussion

Within the endocannabinoid system (ECS), CB1 has received much attention as an important component of the signaling network, regulating many biological responses in various organs as well as the nervous system [26]. GPR119 is a novel endocannabinoid receptor that is mainly expressed in the pancreas and gastrointestinal tract, and has been reported to contribute to glucose homeostasis by stimulating GLP-1 secretion in the rodent brain [27].

Activation of GPR119 has been reported to increase GLP-1 [10,28]. This is known to have beneficial effects on glucose homeostasis by stimulating the insulin secretion of pancreatic beta cells, reducing plasma glucagon, reducing food intake, and delaying gastric emptying [10,29]. In addition, GLP-1 has also been reported to have important anti-inflammatory properties with potential for treating chronic inflammatory diseases, such as type 1 and type 2 diabetes mellitus, asthma, psoriasis, and neurodegenerative disorders [30].

Recently, there has been growing interest in the effect of gut microbiota on the gut and brain. However, few studies have examined how gut microbes affect brain health through the endocannabinoid system.

N-Palmitoyl serinol has been reported as a metabolite in the human microbiome [9]. It is structurally simple and can be chemically synthesized. In previous reports, synthesized N-palmitoyl serinol was found to act as an agonist of CB1 and an endocannabinoid receptor, and to improve skin barrier function by promoting ceramide synthesis [19].

As N-palmitoyl serinol acts as an agonist for GPR119, we aimed to confirm whether it has a similar neuroprotective effect to the GLP-1 family of proteins that has recently attracted attention [31].

In our in vitro tests, NEUROMIDE did not show toxic effects up to 30 μM. In the case of the H_2_O_2_ ROS system, cell viability decreased, and groups pretreated with low (1.2 μM) and medium dosages (6 µM) of NEUROMIDE showed statistically significant increases in cell viability compared with the negative control group. In the case of 30 μM, cell viability decreased with no statistical significance. According to our previous study [19], ceramide synthesis increased by about 20% due to NEUROMIDE (PS, 25 μM) alone. The ROS system also can increase ceramide levels [32]. 

In this experimental system, stress caused by ROS greatly increased ceramide syn-thesis. NEUROMIDE (30 μM) also increased de novo ceramide synthesis. The increased total ceramide from ROS + NEUROMIDE (30 μM) may decrease cell viability slightly.

The ROS-exposed system also showed an increase in inflammatory cytokines such as IL-1β [33]. In line with previous research suggesting that GPR119 agonists decrease in-flammatory cytokines [10], the treatment groups showed dose-dependent decreases in IL-1β gene expression levels. The ROS-exposed system showed dose-dependent increases in acetylcholine for all cases of pretreatment with different NEUROMIDE concentrations, in comparison with the hydrogen-peroxide-treated group. These in vitro results suggest that NEUROMIDE can have a positive effect in protecting nerve cells from damage caused by oxidative stress.

To measure the effectiveness of NEUROMIDE in an in vivo system, we used scopolamine-treated mice as the neurodegenerative disease model. In the case of N-palmitoylethanolamide (PEA), which is very similar structure with NEUROMIDE, 3~30 mg/kg/day showed neuroprotective effects on an Alzheimer model [34]. In our experiment, we aimed to investigate the effectiveness of 16~100 mg/kg/day to assess toxicity or other side effects at higher concentrations of PEA. 

After daily oral administration of NEUROMIDE for 4 weeks, a passive avoidance test was performed, and levels of acetylcholine, AchE activity, IL-1β, and TNF-α in the brain were compared with those in the scopolamine-treated group. Scopolamine-treated mice have already been shown to have elevated levels of inflammatory cytokines (e.g., TNF-α, IL-1β, and IL-18) and cognitive impairment [35].

After daily oral administration of NEUROMIDE for 5 weeks, the Morris water maze task was performed. Mice administered NEUROMIDE showed increased swimming time and distance in the quadrant in which the platform was hidden, compared with mice treated with only scopolamine, suggesting that NEUROMIDE had an effect in improving memory.

To observe and quantify cognitive impairment in the mice, we performed a series of passive avoidance tests. After administration of scopolamine to induce a decline in memory and learning ability, an electric shock was applied. The average latency time for the scopolamine-treated group was 7 s, which was significantly shorter than the average of 205.9 s for the vehicle control group. This shows that the cognitive function of the mice was heavily impaired by scopolamine. Furthermore, the acetylcholine levels decreased and the levels of TNF-α and IL-1β increased.

When treated with NEUROMIDE, however, the latency times increased from 7 s (sco-polamine treated) to 35.7 s (100 mg/kg NEUROMIDE) in a dose-dependent manner. TNF-α content in the hippocampus decreased from 239 pg/mg tissue protein (scopolamine treated) to 189 pg/mg tissue protein (100 mg/kg NEUROMIDE), dose dependently. IL-1β content decreased from 214 pg/mg tissue protein (scopolamine treated) to 151 pg/mg tissue protein (100 mg/kg NEUROMIDE). Acetylcholine levels increased from 0.9 mmol/mg protein (scopolamine treated) to 1.1 mmol/mg protein (16 mg/kg), 1.1 mmol/mg protein (40 mg/kg), and 1.5 mmol/mg protein (100 mg/kg).

The experiments performed in this study aimed to determine the neuroprotective potential of NEUROMIDE for treating cognitive impairment and memory impairment, which were induced by scopolamine in vivo and H_2_O_2_-induced oxidative stress in vitro. 

In our previous study, NEUROMIDE was found to activate CB1R and improve skin barrier recovery [17], and it has shown effectiveness in an atopic dermatitis model, representative of inflammatory skin disease [18]. 

**Figure 8:**
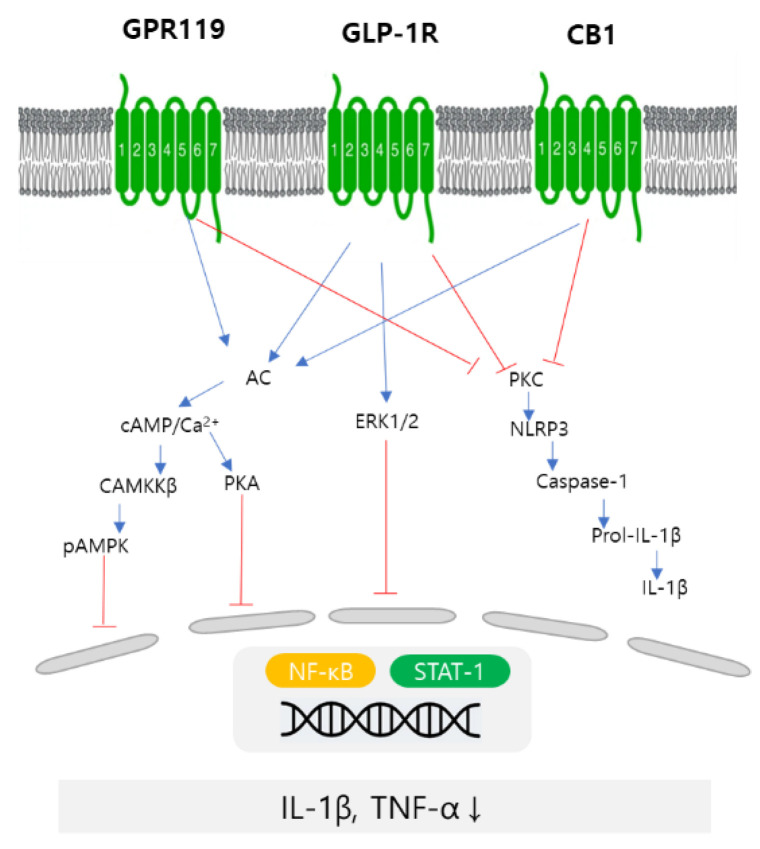
A proposed scheme of the pathway by which NEUROMIDE can inhibit the expression of inflammatory cytokines.

It has been reported that the activation of GRP119 increases the level of GLP-1 in the blood and is ultimately beneficial for glucose homeostasis [10]. In addition to benefits associated with GPR119, research results suggest that GLP-1-based therapies also show anti-inflammatory effects in chronic inflammation. In Figure 8, the pathway is proposed by which the endocannabinoid receptor agonist lowers the levels of proinflammatory cytokines. In this experiment, NEUROMIDE showed effectiveness in decreasing IL-1β in ROS-stressed PC-12 cells, and IL-1β and TNF-α in scopolamine-treated mice.

There seem to be no clear research results to confirm whether inflammatory cytokines increase the activity of acetylcholinesterase, but it is known that when the levels of inflammatory cytokines increase, the amount of acetylcholine decreases [36]. In our experiment, NEUROMIDE reduced the levels of inflammatory cytokines. NEUROMIDE showed inhibition of acetylcholine esterase compared with scopolamine-treated group, albeit not dose-dependently, and the level of acetylcholine in-creased compared with the scopolamine group.

Recently, there has been rising interest in the effect of the gut microbiota on the gut and the brain. However, few studies have examined how gut microbes affect brain health. NEUROMIDE, with bio-identical structure to the gut microbiome’s metabolite, has been reported as an agonist of CB1 and GPR119 [9,17]. In this experiment, NEUROMIDE showed effectiveness in reducing inflammatory cytokines and increasing levels of acetylcholine in the PC-12/ROS system and scopolamine cognitive impairment system. In this experiment, NEUROMIDE showed some mitigation of cognitive decline, but further study is necessary to verify the direct consequences of the reduction of inflammatory cytokines, and the mechanism by which cognitive decline is mitigated through the endocannabinoid system. Toxicology tests will be needed to confirm these results, to determine the safety of NEUROMIDE in oral administration and its effectiveness in healthy subjects.

## Data Availability

Original data are available from Y.S. (yhseo@dtncro.co.kr).

## References

[1] Devane W.A., Hanus L., Breuer A., Pertwee R.G., Stevenson L.A., Griffin G., Gibson D., Mandelbaum A., Etinger A., Mechoulam R. (1992). Isolation and structure of a brain constituent that binds to the cannabinoid receptor. Science.

[2] Sharir H., Console-Bram L., Mundy C., Popoff S.N., Kapur A., Abood M.E. (2012). The Endocannabinoids Anandamide and Vi-rodhamine Modulate the Activity of the Candidate Cannabinoid Receptor GPR55. J. Neuroimmune Pharmacol..

[3] Lauffer L.M., Iakoubov R., Brubaker P.L. (2009). GPR119 Is Essential for Oleoylethanolamide-Induced Glucagon-Like Peptide-1 Secretion from the Intestinal Enteroendocrine L-Cell. Diabetes.

[4] Gonsiorek W., Lunn C., Fan X., Narula S., Lundell D., Hipkin R.W. (2000). Endocannabinoid 2-arachidonyl glycerol is a full agonist through human type 2 cannabinoid receptor: Antagonism by anandamide. Mol. Pharmacol..

[5] Hansen K.B., Rosenkilde M.M., Knop F.K., Wellner N., Diep T.A., Rehfeld J.F., Andersen U.B., Holst J.J., Hansen H.S. (2011). 2-Oleoyl Glycerol Is a GPR119 Agonist and Signals GLP-1 Release in Humans. J. Clin. Endocrinol. Metab..

[6] https://hmdb.ca/metabolites/HMDB0013654.

[7] https://foodb.ca/compounds/FDB029624.

[8] Bieberich E., Kawaguchi T., Yu R.K. (2000). N-Acylated Serinol Is a Novel Ceramide Mimic Inducing Apoptosis in Neuroblastoma Cells. J. Biol. Chem..

[9] Cohen L.J., Esterházy D., Kim S.-H., Lemetre C., Aguilar R.R., Gordon E.A., Pickard A.J., Cross J.R., Emiliano A.B., Han S.M. (2018). Corrigendum: Commensal bacteria make GPCR ligands that mimic human signalling molecules. Nature.

[10] Zhao J., Zhao Y., Hu Y., Peng J. (2021). Targeting the GPR119/incretin axis: A promising new therapy for metabolic-associated fatty liver disease. Cell. Mol. Biol. Lett..

[11] Im D.-S. (2021). GPR119 and GPR55 as Receptors for Fatty Acid Ethanolamides, Oleoylethanolamide and Palmitoylethanolamide. Int. J. Mol. Sci..

[12] Suardíaz M., Estivill-Torrús G., Goicoechea C., Bilbao A., Rodríguez de Fonseca F. (2007). Analgesic properties of oleoylethanola-mide (OEA) in visceral and inflammatory pain. Pain.

[13] Tough I.R., Forbes S., Herzog H., Jones R.M., Schwartz T.W., Cox H.M. (2018). Bidirectional GPR119 Agonism Requires Peptide YY and Glucose for Activity in Mouse and Human Colon Mucosa. Endocrinology.

[14] Cully M. (2017). Gut feeling on bacterial GPCR agonists. Nat. Rev. Drug Discov..

[15] Markovics A., Angyal Á., Tóth K.F., Ádám D., Pénzes Z., Magi J., Pór Á., Kovács I., Törőcsik D., Zouboulis C.C. (2020). GPR119 Is a Potent Regulator of Human Sebocyte Biology. J. Investig. Dermatol..

[16] Scott G.A., Jacobs S.E., Pentland A.P. (2006). sPLA2-X Stimulates Cutaneous Melanocyte Dendricity and Pigmentation Through a Lysophosphatidylcholine-Dependent Mechanism. J. Investig. Dermatol..

[17] Jeong S., Kim M.S., Lee S.H., Park B.D. (2019). Epidermal Endocannabinoid System (EES) and its Cosmetic Application. Cosmetics.

[18] Wen S., Ye L., Liu D., Yang B., Man M.Q. (2021). Topical N-palmitoyl serinol, a commensal bacterial metabolite, prevents the de-velopment of epidermal permeability barrier dysfunction in a murine model of atopic dermatitis-like skin. Can. J. Vet. Res..

[19] Shin K.-O., Kim S., Park B., Uchida Y., Park K. (2021). N-Palmitoyl Serinol Stimulates Ceramide Production through a CB1-Dependent Mechanism in In Vitro Model of Skin Inflammation. Int. J. Mol. Sci..

[20] Grill M., Högenauer C., Blesl A., Haybaeck J., Golob-Schwarzl N., Ferreirós N., Thomas D., Gurke R., Trötzmüller M., Köfeler H.C. (2019). Members of the endocannabinoid system are distinctly regulated in inflammatory bowel disease and col-orectal cancer. Sci. Rep..

[21] Houser M., Tansey M.G. (2017). The gut-brain axis: Is intestinal inflammation a silent driver of Parkinson’s disease pathogenesis?. NPJ Parkinson’s Dis..

[22] Meseguer Henarejos A.B., Popović N., Bokonjić D., Morales-Delgado N., Alonso A., Caballero Bleda M., Popović M. (2020). Sex and Time-of-Day Impact on Anxiety and Passive Avoidance Memory Strategies in Mice. Front. Behav. Neurosci..

[23] Lee J.-S., Kim H.-G., Lee H.W., Han J.-M., Lee S.-K., Kim D.W., Saravanakumar A., Son C.-G. (2015). Hippocampal memory en-hancing activity of pine needle extract against scopolamine-induced amnesia in a mouse model. Sci. Rep..

[24] Melo J.B., Agostinho P., Oliveira C. (2003). Involvement of oxidative stress in the enhancement of acetylcholinesterase activity induced by amyloid beta-peptide. Neurosci. Res..

[25] Kaneko N., Kurata M., Yamamoto T., Morikawa S., Masumoto J. (2019). The role of interleukin-1 in general pathology. Inflamm. Regen..

[26] Howlett A.C., Blume L.C., Dalton G.D. (2010). CB1 Cannabinoid Receptors and their Associated Proteins. Curr. Med. Chem..

[27] Brown A.J. (2007). Novel cannabinoid receptors. Br. J. Pharmacol..

[28] Lan H., Lin H., Wang C., Wright M., Xu S., Kang L., Juhl K., Hedrick J., Kowalski T. (2011). Agonists at GPR119 mediate secretion of GLP-1 from mouse enteroendocrine cells through glucose-independent pathways. Br. J. Pharmacol..

[29] Overton H.A., Fyfe M.C.T., Reynet C. (2008). GPR119, a novel G protein-coupled receptor target for the treatment of type 2 diabetes and obesity. Br. J. Pharmacol..

[30] Lee Y.-S., Jun H.-S. (2016). Anti-Inflammatory Effects of GLP-1-Based Therapies beyond Glucose Control. Mediat. Inflamm..

[31] Borlongan C.V., Esparza-Salazar F.D.J., Lezama-Toledo A.R., Rivera-Monroy G. (2021). Exendin-4 for Parkinson’s disease. Brain Circ..

[32] Abrahan C.E., Miranda G.E., Agnolazza D.L., Politi L.E., Rotstein N.P. (2010). Synthesis of Sphingosine Is Essential for Oxidative Stress-Induced Apoptosis of Photoreceptors. Investig. Ophthalmol. Vis. Sci..

[33] Li P., Li P. (2015). Neuroprotective effect of paeoniflorin on H_2_O_2_-induced apoptosis in PC12 cells by modulation of reactive oxygen species and the inflammatory response. Exp. Ther. Med..

[34] D’agostino G., Russo R., Avagliano C., Cristiano C., Meli R., Calignano A. (2012). Palmitoylethanolamide Protects against the Amyloid-β25-35-Induced Learning and Memory Impairment in Mice, an Experimental Model of Alzheimer Disease. Neuropsychopharmacology.

[35] Cheon S.Y., Koo B.-N., Kim S.Y., Kam E.H., Nam J., Kim E.J. (2021). Scopolamine promotes neuroinflammation and delirium-like neuropsychiatric disorder in mice. Sci. Rep..

[36] Reale M., De Angelis F., Di Nicola M., Capello E., Di Ioia M., de Luca G., Lugaresi A., Tata A.M. (2012). Relation between pro-inflammatory cytokines and acetylcholine levels in relapsing-remitting multiple sclerosis patients. Int. J. Mol. Sci..

